# Oocyte-Secreted Serum Biomarkers GDF9 and BMP15 in Women with Endometriosis

**DOI:** 10.1007/s43032-022-01107-6

**Published:** 2022-11-15

**Authors:** Aiat Shamsa, Robert B. Gilchrist, David M. Robertson, Rachael J. Rodgers, Mark W. Donoghoe, William L. Ledger, Jason A. Abbott, Angelique H. Riepsamen

**Affiliations:** 1 Clinical School of Medicine, University of New South Wales, Sydney, NSW Australia; 2 Royal Hospital for Women, Randwick, NSW Australia; 3 Stats Central, Mark Wainwright Analytical Centre, University of New South Wales, Sydney, NSW Australia

**Keywords:** Oocyte-secreted factors, Endometriosis, Biomarker, GDF9, BMP15

## Abstract

Oocyte-secreted growth differentiation factor 9 (GDF9) and bone morphogenetic protein 15 (BMP15) are critical paracrine regulators of female fertility. Recent studies demonstrated that serum concentrations are associated with the number of oocytes retrieved during IVF, and therefore potential clinical use as biomarkers. However, it is unknown if the presence of endometriosis affects serum GDF9 or BMP15. An exploratory case–control study was prospectively performed on 60 women who underwent laparoscopy between April 2017 and August 2018 at two hospitals. GDF9 and BMP15 were measured by validated immunoassays in pre-operative serum samples. Data were analysed relative to laparoscopic assessment of endometriosis and staging. There were 35 women with confirmed laparoscopic diagnosis of endometriosis and 25 controls with no evidence of endometriosis at laparoscopy. GDF9 was detectable in 40% of controls and 48% of cases. There was no difference in median GDF9 concentrations between controls (20.0 pg/ml, range 20.0–2504 pg/ml) and cases (20.0 pg/ml, range 20.0–2963 pg/ml). BMP15 was detectable in 48% of controls and 58% of cases, with no difference in median concentrations between controls (26.5 pg/ml, range 24.0–1499 pg/ml) and cases (24.0 pg/ml, range 24.0–796 pg/ml). Furthermore, there were no significant differences in the proportion of detectable samples or concentrations of GDF9 or BMP15 with differing severities of endometriosis. In conclusion, serum concentrations of oocyte-secreted factors, GDF9 and BMP15 did not differ between control patients and patients with endometriosis. For clinical application in reproductive medicine, GDF9 and BMP15 serum biomarker quantitation is unlikely to be aberrant in the presence of endometriosis.

## Introduction

Endometriosis is a chronic oestrogen-dependant gynaecological condition, presenting with persistent pelvic pain symptoms and infertility [1]. One in nine women of reproductive age will be diagnosed with endometriosis [2]. Both environmental and genetic factors contribute to the development of endometriosis [3, 4] and an improved understanding of aetiology will enable an increase in diagnostic, therapeutic and preventive strategies to reduce the substantive physical and economic burden of endometriosis [3, 5, 6].

Symptoms, clinical examination and imaging aid the diagnosis of endometriosis; however, currently the internationally accepted standard of diagnosis is direct visualisation of endometriosis with or without histology at laparoscopic surgery [7]. Whilst many studies have investigated biomarkers for non-invasive diagnosis of endometriosis, no single biomarker or combinations of biomarkers have proven sufficiently sensitive and specific to replace the more invasive laparoscopic surgery [8, 9]. Serum markers investigated include growth factors involved in angiogenesis, cell-adhesion molecules, DNA-repair molecules, endometrial and mitochondrial proteome arrays, hormonal, inflammatory, myogenic, neural and tumour markers, as well as the ovarian markers anti-Müllerian hormone (AMH) and follicle-stimulating hormone (FSH) (reviewed in 9, and 10).

As endometriosis has been reported to be associated with infertility [11, 12], oocyte-secreted proteins could be altered in women with endometriosis and thus be potential biomarkers for diagnosis. Growth-differentiation factor 9 (GDF9) and bone morphogenetic factor 15 (BMP15) are two oocyte-secreted molecules that play a central role in ovarian function and oocyte quality [13]. GDF9 and BMP15 are secreted by the oocyte and regulate the quality of the egg by interacting with the surrounding somatic cells [13]. It is thus expected that GDF9 and BMP15 concentrations in human serum will vary with differing reproductive pathologies [14], and recently we demonstrated evidence of an association between serum GDF9 and the number of oocytes retrieved during IVF [15]. Mutations that reduce or disrupt the production of these oocyte-secreted factors have been observed in conditions associated with subfertility [14] including polycystic ovary syndrome (PCOS), primary ovarian insufficiency (POI) and Turners syndrome [16–18]. An indication that aberrant GDF9 function may be associated with endometriosis arises from reports of lower concentrations of GDF9 protein and mRNA in follicular fluid of patients with severe endometriosis [19, 20]. However, until recently [15], there have not been assay methods available to reliably measure GDF9 or BMP15 in human sera and thus assess if concentrations are altered in patients with endometriosis. This study aims to measure serum GDF9/BMP15 in patients with and without endometriosis, to assess whether concentrations are affected by the presence and/or stage of endometriosis, diagnosed by laparoscopic procedures.

## Methods

This was a prospective cohort study of 60 women, approved by the institutional ethical committee (South Eastern Sydney Local Health District ethics committee approval: 17/106), with all participants providing written informed consent. Patients aged 18 to 45 years undergoing planned gynaecological laparoscopy between April 2017 and August 2018 at two university affiliated hospitals in Sydney, Australia, were involved in the study. Exclusion criteria were a previous history of cancer/chemotherapy, previous or current disease receiving gonadotoxic therapies, or inclusion in concurrent studies.

### Serum Sampling

Blood samples were drawn immediately pre-operative by venepuncture on sites contralateral to the upper extremity receiving IV fluid therapy. All blood samples were collected in serum-separating tubes (BD, Australia), allowed to clot at room temperature for 30 min to 2 h following collection, then centrifuged and sera frozen. Frozen sera were defrosted, aliquoted for single use and stored at − 80 °C prior to analysis.

### Serum Analysis

An ELISA for detecting BMP15 in human serum was previously developed and validated [15], using a monoclonal antibody directed at the mature region of the protein (supplied by the Oxford Brooks University, UK), and recombinant promature human BMP15 protein supplied by Associate Professor Craig Harrison at Monash University (Clayton, Australia). The BMP15 assay sensitivity was 24 pg/ml, with intra- and inter-assay variations of 5.5% and 10.2%, respectively. Serum GDF9 was measured using a commercially available GDF9 ELISA as per the manufacturer’s protocol (AL-176; Lot #053118; Ansh Labs, Webster, TX, USA), assayed as singletons [21]. The Ansh Labs GDF9 ELISA used antibodies directed at the mature region of GDF9 [22]. The GDF9 assay sensitivity was 20.0 pg/ml, with intra- and inter-assay variations of 2% and 8%, respectively. Assay sensitivity, or limit of quantification (LOQ), was defined as the dose of the standard corresponding to 2X SD absorbance units above the assay blank value. Samples that gave readings less than the sensitivity value were considered undetectable and given a value equal to the sensitivity value.

Serum anti-Müllerian hormone (AMH) was assayed using the Elecsys AMH Plus assay on the Cobas® e411 automated platform, with an electro chemiluminescent (ECL) method principle analyser (Roche Diagnostics, North Ryde, NSW, Australia). The assays were undertaken at the South Eastern Area Laboratory Services, Prince of Wales Hospital (Randwick, NSW).

### Clinical Data Collection

Demographic information, past medical, surgical, gynaecological and family histories for all patients were recorded. All study data were entered onto and managed using Research Electronic Data Capture (REDCap) software [23].

### Surgical Reporting

All patients underwent planned gynaecological laparoscopy, which included but was not limited to diagnostic surgery for the investigation of pain and/or fertility, excision of endometriosis, ovarian cystectomy and hysterectomy. The revised American Society of Reproductive Medicine (rASRM) 1996 classification of endometriosis score was employed. Endometriosis was excised and diagnosis was confirmed by histology. Patients with no evidence of endometriosis at laparoscopy were assigned to the control group.

### Statistical Analyses

Statistical analyses of patient demographics and gynaecological history were performed using the Statistical Package STATA 12.0 (StataCorp. 2011. Stata Statistical Software: Release 12. College Station, TX: StataCorp LP). Categorical variables were compared using a chi-square test. For continuous variables, the normality assumption was checked using the D’Agostino and Pearson test for normality. Variables that were approximately normally distributed were evaluated using independent *t*-tests, whilst those that were non-normal were compared using a Mann–Whitney *U* test.

The statistical package R version 4.0.4 [24] was used to evaluate the association between biomarkers and the presence of endometriosis, as well as the rASRM score for stage of endometriosis. LOQ for GDF9 and BMP15 assays led to the presence of left-censored observations, where an upper bound on the concentration in a sample was obtained, rather than its actual value. Nevertheless, these observations can be used in maximum likelihood estimation if parametric assumptions can be made about the distribution of these measurements in the population, as we have done previously [15]. To this end, the distributions of each biomarker, as well as AMH, were examined using Q-Q plots accounting for left-censoring [25], which suggested that the observed concentrations approximately followed a log-normal distribution. The survreg function [26] was used to estimate the association between categorical variables and mean concentration of each biomarker, assuming a log-normally distributed outcome with left-censored observations. Geometric means and their 95% confidence intervals (95% CI) were estimated within each group, as well as the ratio of geometric means, and its 95% CI, between groups. A significance level of 5% was used throughout the study.

## Results

Of the 60 patients enrolled, 35 had confirmed intra-operative diagnosis of endometriosis and the 25 without endometriosis at laparoscopy were used as the control group. Demographics of the two groups are reported in Table 1. One third of the cases with endometriosis had evidence of moderate to severe disease, i.e. stage 3–4.

**Table 1:** Demographic characteristics and gynaecological history of participants

	Control (*n* = 25)	Endometriosis (*n* = 35)	*p*
Age, mean ± SD	35.5 ± 8.2	32.4 ± 7.8	0.103
BMI, mean ± SD	27.7 ± 5.6	24.5 ± 4.8	**0.019**
Gravidity, mean ± SD	1.24 ± 1.54	0.71 ± 1.36	0.096
Parity, mean ± SD	0.92 ± 1.32	0.34 ± 0.97	**0.029**
Gravidity, median (range)	1 (0–5)	0 (0–5)	0.096
Parity, median (range)	0 (0–5)	0 (0–4)	**0.029**
Nulliparity, *n* (%)			0.199
Yes	12 (48.0)	22 (64.7)	
No	13 (52.0)	12 (35.3)	
Ethnicity, *n* (%)			0.471
Caucasian	17 (68.0)	20 (58.8)	
Non-Caucasian	8 (32.0)	14 (41.2)	
Tobacco use, *n* (%)			0.116
Ever used	2 (8.0)	8 (23.5)	
Never used	23 (92.0)	26 (76.5)	
Alcohol consumption, *n* (%)			0.223
0–1 standard drink per week	12 (48.0)	11 (32.4)	
> 1 standard drink per week	13 (52.0)	23 (67.6)	
Regularity of cycle, *n* (%)			0.745
Regular	15 (62.5)	22 (66.7)	
Irregular	9 (37.5)	11 (33.3)	
Cycle phase at time of surgery, *n* (%)			**0.037**
Follicular	4 (26.7)	13 (61.9)	
Luteal	11 (73.3)	8 (38.1)	
Hormonal contraception use, *n* (%)			0.411
Yes	8 (33.3)	8 (23.5)	
No	16 (66.7)	26 (76.5)	
History of PCOS, *n* (%)			0.190
Yes	7 (28.0)	5 (14.3)	
No	18 (72.0)	30 (85.7)	

Demographics and characteristics with a boldface *P* are statistically different between cases and controls (*p* value <0.05)

**Fig. 1:**
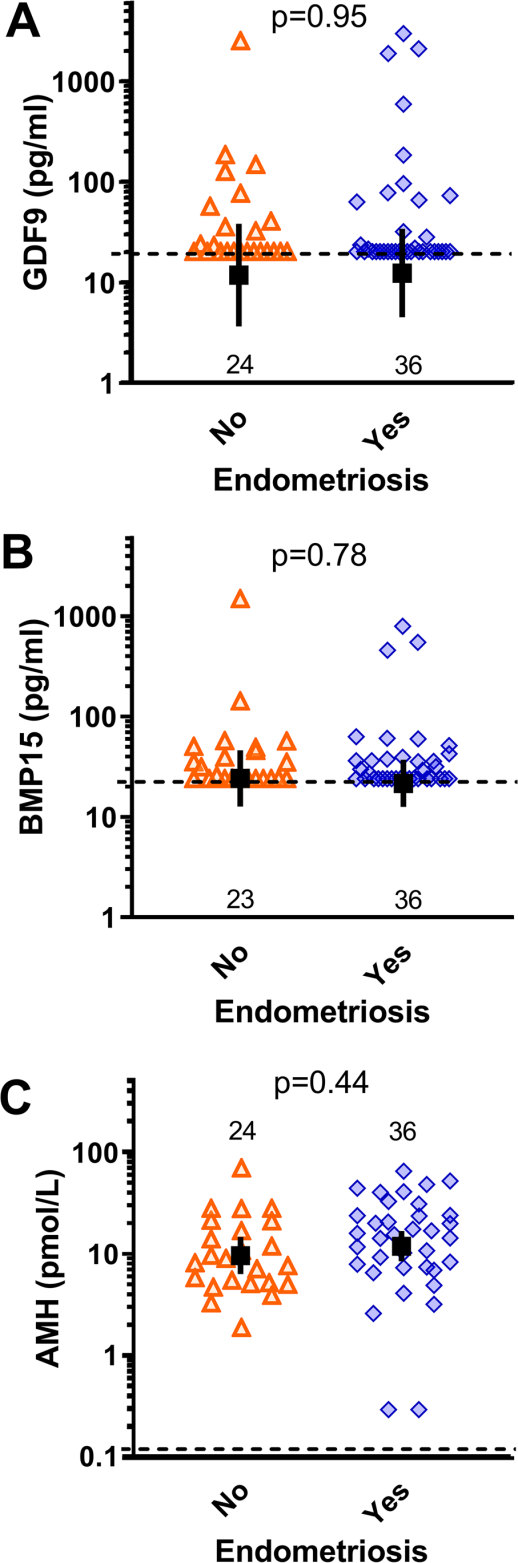
Serum biomarker concentrations in women with and without endometriosis. Dashed horizontal lines indicate the limit of quantification of the assays, black squares with bars represent the estimated geometric ± 95% CI, and numbers above the x-axis show the number of women in each group. *P*-values within panels indicate statistical comparisons between groups

**Fig. 2:**
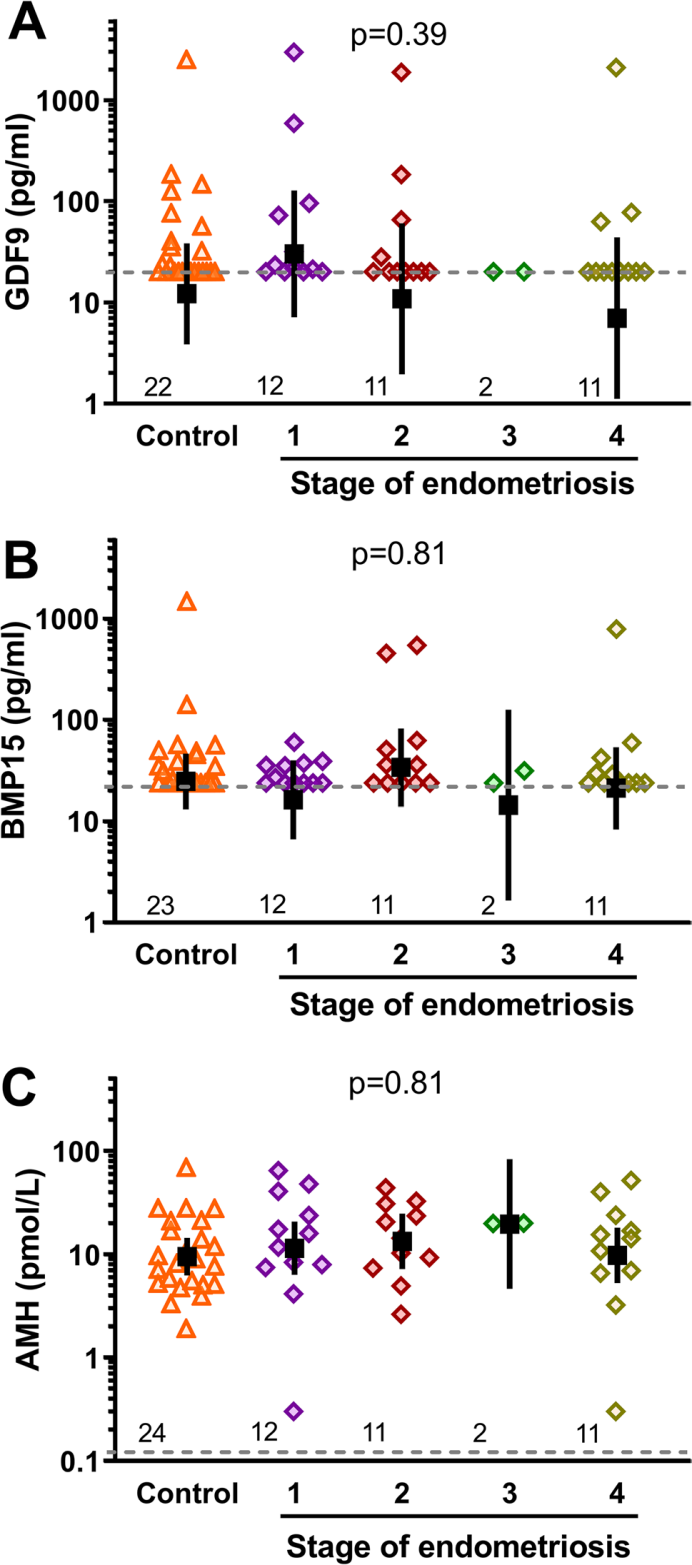
Serum biomarker concentrations and stage of endometriosis. Dashed horizontal lines indicate the limit of quantification of the assays, black squares with bars represent the estimated geometric ± 95% CI, and numbers above the x-axis show the number of women in each group. *P*-values within panels indicate statistical comparisons between groups

There were no significant differences in the mean serum concentrations of GDF9 (Fig. 1A) between controls (11.9 pg/ml, 95% CI: 3.7, 38.5) and patients with endometriosis (12.4 pg/ml, 95% CI: 4.5, 34.2), or in serum BMP15 (Fig. 1B) between controls (24.2 pg/ml, 95% CI: 12.7, 46.1) and patients with endometriosis (21.6 pg/ml, 95% CI: 12.6, 37.2). Concentrations of these oocyte-secreted factors were in the pg/ml range, with 40% of controls and 48% of cases with detectable samples above the LOQ of the GDF9 assay, and 49% of controls and 58% of cases above the LOQ in the BMP15 assay. There were no differences in the proportions of detectable samples between endometriosis and control groups. No statistically significant differences in GDF9 or BMP15 were observed between the different stages of endometriosis (Fig. 2A, B), or the proportion of serum samples with detectable GDF9 or BMP15 with different severities of the disease. Serum BMP15 and GDF9 in all women were also assessed relative to AMH, a widely utilised endocrine indicator of ovarian reserve, and no correlation was found. Serum AMH values also did not differ between controls (9.6 pmol/l, 95% CI: 6.3, 14.6) and patients with endometriosis (11.8 pmol/l, 95% CI: 8.4, 16.7) (Fig. 1C), nor by stage of endometriosis (Fig. 2C).

There were no significant differences in biomarker concentrations when controls were compared to patients with an endometrioma (*n* = 14). The presence of other gynaecological pathogenesis, i.e. adenomyosis (*n* = 3), endosalpingiosis (*n* = 1) and leiomyomas (*n* = 9), did not affect the concentration of GDF9 and BMP15 (data not shown).

## Discussion

Follicle and oocyte quality may be impaired in patients with endometriosis, with inhibitory effects observed on follicle growth, steroidogenesis and granulosa cell function (reviewed in 27). Studies of ovarian reserve markers, AMH, antral follicle count and FSH also suggest a decline in oocyte quantity in patients with endometriosis (reviewed in 12, 28). Furthermore, endometriosis has been associated with a reduced number of mature oocytes retrieved during IVF and in cases of minimal/mild endometriosis (but not moderate/severe) a reduced fertilisation rate was noted (reviewed in 27). GDF9 and BMP15 as potential serum biomarkers of oocyte quantity/quality may therefore be altered in patients with endometriosis. However, in the present study no evidence was found to support this hypothesis. A previous study reported a lower concentration of GDF9 in the follicular fluid of patients with severe endometriosis [19]. Kawabe and colleagues [20] also showed lower GDF9 mRNA expression in granulosa cells of patients with endometriosis compared to controls. However, they did not find differences in GDF9 concentration in follicular fluid from patients with endometriosis. On the other hand, infertile patients with endometriosis were found to have unchanged GDF9 gene expression levels in cumulus cells of antral follicles compared to controls [29]. Although there is limited literature assessing BMP15 in patients with endometriosis, expression studies of granulosa cells report no significant difference in BMP15 mRNA expression in patients with endometriosis compared to controls [30, 31]. Together, these results do not demonstrate strong evidence of GDF9 and BMP15 being affected by the presence of endometriosis.

A limitation of this study was the large proportion of patients with undetectable concentrations of GDF9 and BMP15 in serum. This may be expected as these are oocyte paracrine factors that are exclusively secreted by oocytes and therefore appear in the circulation in very low concentrations and thus cannot always be readily detected using existing ELISAs. These findings are consistent with recent studies [15, 21] and may explain differences with other studies which reported an association between these markers and endometriosis in follicular fluid using semi-quantitative western blotting, such as Kawabe et al. reporting significant reduction in GDF9 mRNA expression in granulosa cells of women with moderate to severe endometriosis [19, 20]. It is also possible that the assays are not detecting all forms of the circulating proteins, although the assays target the mature regions of GDF9 and BMP15, and the mature dimers are the forms in which these proteins bind to their signalling receptors [32]. It is possible that GDF9/BMP15 may be aberrant in specific populations of women with endometriosis, such as has been reported for AMH relative to endometriomas [33, 34]. Furthermore, in this study, there appears to be no significant difference in the concentration of the biomarkers in different phases of the menstrual cycle. However, one of the limitations of our study is that the sera were drawn immediately pre-operative, irrespective of menstrual cycle stage. A study analysing serum concentrations of GDF9 and BMP15 in ovulatory women with regular cycles reported no statistically significant changes across the menstrual cycle [21]. However, our results may have been more consistent if our serum samples had been collected only in the late follicular phase. The dilutional effect on GDF9 and BMP15 in serum may be important given that their normal concentration is very low and undetectable in a large proportion of women, potentially affecting the lack difference in the biomarker concentrations detected in women with endometriosis and in controls.

This exploratory study demonstrated no significant difference in serum GDF9 and BMP15 concentrations between women with and without laparoscopically diagnosed endometriosis, suggesting that serum measurements of these oocyte-secreted proteins are unlikely to be affected by the presence of endometriosis or to be useful as a diagnostic test for endometriosis. Therefore, for clinical application of GDF9 and BMP15 as potential serum biomarkers in reproductive medicine, quantitation in serum is unlikely to be aberrant due to the presence of endometriosis. A limited number of other histologically diagnosed pathologies, i.e. adenomyosis, endosalpingiosis and leiomyomas, were present in this cohort and their presence did not affect the biomarker concentrations. Hence, it is unlikely that these conditions have affected our results. Furthermore, the novel assays utilised in this study were recently developed and validated [15] and the development of more sensitive assays of GDF9 and BMP15, with lower detection limits, and characterisation of the native forms of GDF9 and BMP15 are needed to understand these biomarkers in different gynaecological disorders and infertility.
